# The effect of *Allium sativum* on ischemic preconditioning and ischemia reperfusion induced cardiac injury

**DOI:** 10.4103/0253-7613.45152

**Published:** 2008

**Authors:** Rajbir Bhatti, Kushlinder Singh, M.P.S. Ishar, Jatinder Singh

**Affiliations:** Department of Pharmaceutical Sciences, Guru Nanak Dev University, Amritsar, India; 1 Department of Pharmacology, Govt. Medical College, Amritsar, India

**Keywords:** Ischemia, ischemic preconditioning, reperfusion

## Abstract

In the present study, the effect of garlic (*Allium sativum*) extract on ischemic preconditioning and ischemia-reperfusion induced cardiac injury has been studied. Hearts from adult albino rats of Wistar strain were isolated and immediately mounted on Langendorff's apparatus for retrograde perfusion. After 15 minutes of stabilization, the hearts were subjected to four episodes of 5 min ischemia, interspersed with 5 min reperfusion (to complete the protocol of ischemic preconditioning), 30 min global ischemia, followed by 120 min of reperfusion. In the control and treated groups, respective interventions were given instead of ischemic preconditioning. The magnitude of cardiac injury was quantified by measuring Lactate Dehydrogenase and creatine kinase concentration in the coronary effluent and myocardial infarct size by macroscopic volume method. Our study demonstrates that garlic extract exaggerates the cardio protection offered by ischemic preconditioning and per se treatment with garlic extract also protects the myocardium against ischemia reperfusion induced cardiac injury.

## Introduction

Ischemic injury is one of the prime causes of cardiovascular mortality and in several clinical situations, early reperfusion has shown to improve myocardial pump function.[1] However, reperfusion has its own inherent limitation by inflicting reperfusion injury.[2] An established determinant of clinical complications and patient survival in an event of acute coronary occlusion is the myocardial infarct size.[3]

Repeated short episodes of ischemia make the myocardium resistant to the deleterious effects of a more prolonged ischemic episode.[4] This paradoxical form of myocardial adaptation has been termed as ischemic preconditioning, which can be experimentally induced by a variety of protocols in different animal species. Ischemia and reperfusion of 5 min duration is as effective as multiple ischemia reperfusion episodes of the same duration, in order to provide protection in dog and rabbit hearts.[5,6] Two episodes of 2 min ischemia and 2 min reperfusion produced the same protection to rabbit heart, as provided by single 5 min occlusion and 5 min reperfusion.[7] Three episodes of 2 min or single episode of 3 min coronary occlusion and reperfusion have been found to be sufficient to prevent arrhythmias due to ischemia reperfusion injury.[8,9]

Allium sativum (Liliaceae) or garlic is a hardy perennial bulbous scapigerous herb, with a flat stem. The lower portion of the plant forms a bulb, which consists of several smaller buds called cloves, surrounded by a thin white or pinkish sheath. The leaves are flat, narrow green; the heads bear small white flowers and bulbils. Since ancient days, it has been cultivated throughout India, Pakistan, Bangladesh, and most tropical countries. The bulbs are known to be antidiabetic, anti-inflammatory, anticancerous, and effective in rheumatism.[10] Raw garlic decreases the levels of glucose, cholesterol, and phospholipids, and is useful in meningitis, rickettsia. It is given with common salt in nervous diseases and headache. The juice relieves earache, bronchitis, gangrene of the lung, whooping cough, laryngeal and pulmonary tuberculosis and duodenal ulcer. In external application, the juice is used as rubifacient, in skin diseases, as an eye drop and in earache.[10]

Garlic is a rich source of organic sulphur compounds, showing a variety of biological activities. The chemical constituents from garlic cloves (bulbs) vary with the isolation procedure. Garlic bulbs contain up to 1% of fresh weight, S-allylcysteine-S-oxide called alliin, which is converted into oxide of diallyl disulphide called allicin, by contact with enzyme allinase liberated during tissue injuries.[11] Extraction of garlic with ethyl alcohol at room temperature yields allicin.[12] Extraction of garlic with ethanol at subzero temperature yields an odorless amino acid alliin.[11]

## Materials and Methods

### Preparation of garlic extract

Five hundred grams of garlic cloves, with the outer coat removed, were crushed in a grinder (Philips) and soaked in 1 liter of 95% ethanol for 72 hours. Thereafter, the solvent was evaporated under reduced pressure in a rota-vapouriser (Laborota 4001, Heidolph), to obtain a viscous extract. The yield was 24.6 g. For administration, the extract was suspended in 0.5% dimethyl sulfoxide (DMSO) and diluted with distilled water. This extract was added to the Kreb-Henseliet's solution to give a concentration of 0.5%.

### Global ischemia-reperfusion in isolated perfused rat heart

Wistar albino rats (125-150g) of both sexes were employed in the present study. The animals were kept in 12 h light and 12 h dark cycle, and were fed on standard laboratory chow. They had free access to tap water. Heparin (500 I.U., i.p.) was administered 20 min before sacrificing the animal by stunning. The heart was rapidly excised and immediately mounted on Langendorff's apparatus.[13] The aorta was perfused at a constant pressure of 70 mm Hg, with Krebs-Henseleit buffer (NaCl 118 mM; KCl 4.7 mM; CaCl_2_ 2.5 mM; Mg SO_4_. 7H_2_O 1.2 mM; NaHCO_3_ 25 mM; KH_2_PO_4_ 1.2 mM; C_6_H_12_O_6_ 11mM), pH 7.4, maintained at 37°C bubbled with 95 % O_2_ and 5% CO_2_. The flow rate was maintained at 6-9 ml/min, using Hoffman's screws. The heart was enclosed by a double walled jacket, the temperature of which was maintained by circulating water heated to 37°C. Global ischemia was produced for 30 min, by blocking the in-flow of Kreb's buffer. It was followed by reperfusion for 120 min. Coronary effluent was collected after stabilization (Basal), before global ischemia (BGI), immediately after reperfusion (immediately), 5 min, 15 min and 30 min after reperfusion, for lactate dehydrogenase (LDH) and Creatine kinase (CK)estimation.

### Infarct size measurement

Infarct size was measured by macroscopic method and the infracted area reported as the percentage of total ventricular area.[14] The hearts were removed from the Langendorff's apparatus and both the auricles and the root of the aorta were excised, and the ventricles were frozen. These were then sliced into uniform sections of 2-3 mm thickness and incubated in 1% triphenyltetrazolium chloride (TTC), at 37° C in 0.2M Tris buffer (pH 7.4), for 20 minutes. Triphenyltetrazolium chloride was converted to red formazone pigment by reduced Nicotinamide Adenine Dinucleotide (NADH) and dehydrogenase enzyme and, therefore, stained the viable cells deep red, while the infracted cells remained unstained or dull yellow. The ventricular slices were placed between two glass plates and a transparent plastic grid with 100 squares in 1 cm^2^ was placed above it. The average area of each slice was calculated by counting the number of squares on either side and similarly the non stained dull yellow area was counted. The infracted area was expressed as a percentage of the total ventricular area.

### Estimation of lactate dehydrogenase (LDH)

Estimation of lactate dehydrogenase (LDH) was estimated in coronary effluent, using the 2,4-DNPH method, as described by King.[15]

### Estimation of creatine kinase (CK)

creatine kinase (CK) was measured in the coronary effluent, using the modified method of Hughes.[16]

### Experimental protocols

Six groups of Wistar albino rats were employed in the present study. In each group, the isolated rat heart was allowed to stabilize for 20 minutes and all the preparations were perfused with K-H (Krebs-Henseleit) solution, during the stabilization period.

Group I (Control group; n = 5): Isolated rat heart was perfused for 40 min with K-H solution and then subjected to 30 min global ischemia, followed by 120 min of reperfusion.

Group II (Garlic extract treated group; n = 5): Isolated rat heart was perfused with K-H solution containing ethanol extract of garlic and then subjected to 30 min global ischemia, followed by 120 min reperfusion with K-H solution containing garlic extract.

Group III (Ischemic preconditioned group; n = 5): After stabilization, the hearts were subjected to four episodes of 5 min ischemia and 5 min reperfusion with K-H solution and then subjected to 30 min global ischemia and 120 min of reperfusion.

Group IV (Garlic in Ischemic preconditioned group; n = 5): After stabilization, isolated rat hearts were subjected to four episodes of 5 min ischemia and 5 min reperfusion, with K-H solution containing garlic extract, and then subjected to 30 min global ischemia, followed by 120 min of reperfusion with KH solution.

Group V (Garlic preconditioned group; n=5): After stabilization, the hearts were subjected to four episodes of 5 min perfusion with KH solution containing garlic extract, interspersed by 5 min perfusion with KH solution containing no drug. This was followed by 30 min global ischemia and reperfusion with K-H solution for 120 min.

Group VI (Vehicle treated group; n = 5): After stabilization, the isolated rat hearts were perfused with K-H solution containing vehicle for 40 min and then subjected to global ischemia and 120 min of reperfusion with K-H solution containing the vehicle.

### Statistical analysis

Values for enzymatic data and infarct size were expressed as mean ± SEM. Statistical significance was calculated using one-way analysis of variance, followed by Duncan's test as *post hoc* test. A value of *P*<0.05 was considered to be statistically significant.

### Drugs and chemicals

Garlic extract was prepared in ethanol (95%). Tris buffer (0.2 M, pH 7.4) was prepared by dissolving 2.42 g of tris (Qualigens, Mumbai, India) in 82 ml of 0.2 M HCl and the volume was made up to 100ml with distilled water. One percent triphenyltetrazolium chloride (TTC) was prepared by dissolving 1 g of TTC (Thomas Baker Chemicals, Mumbai, India) in 100 ml of 0.2 M tris buffer. All other reagents used in the study were analytical grade of Qualigens (Glaxo, Mumbai, India), Sisco Research Laboratories (Mumbai, India) and Central Drug House (New Delhi, India).

## Results

### Effect of preconditioning and garlic extract on ischemia reperfusion induced LDH release

**Figure 1:**
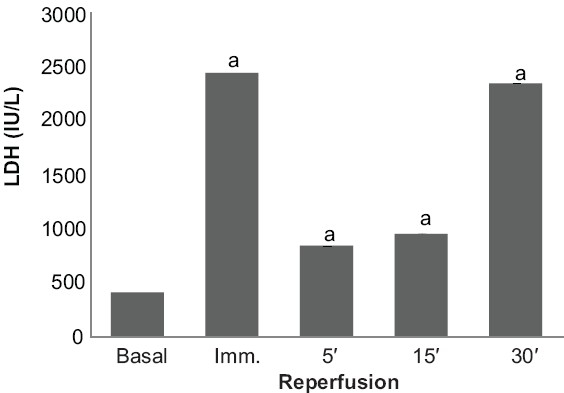
The effect of ischemia and reperfusion on the release of Lactate Dehydrogenase (LDH) in the isolated rat heart. a < 0.05 vs basal

**Figure 2:**
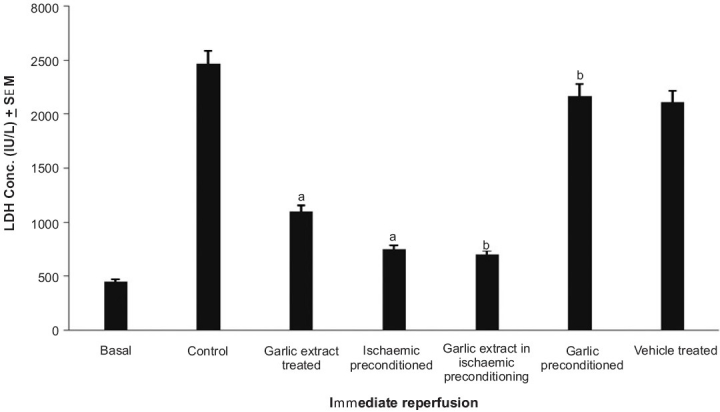
The effect of preconditioning and garlic extract on ischemia reperfusion induced lactate dehydrogenase (LDH) release, immediately after reperfusion. a = *P*<0.05 vs basal; b = *P*<0.05 vs control; c = *P*<0.05 vs ischemia preconditioned

**Figure 3:**
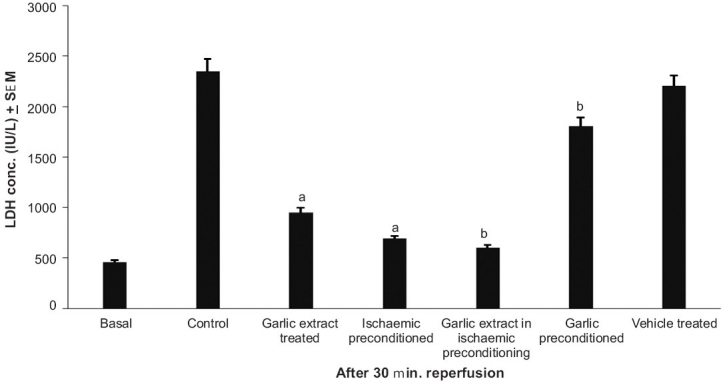
The effect of preconditioning and garlic extract on ischemia reperfusion induced lactate dehydrogenase (LDH) release after 30 min of reperfusion. a = *P*<0.05 vs basal; b = *P*<0.05 vs control; c = *P*<0.05 vs ischemia preconditioned

The peak release of LDH in coronary effluent of isolated rat heart was observed immediately and 30 min after reperfusion [Figure 1]. Ischemic preconditioning and garlic treatment showed significant decrease in LDH release noted immediately and 30 min after reperfusion, as compared to the control group; whereas, garlic preconditioning and vehicle treatment did not significantly alter the profile as compared to the control. Garlic extract administered during ischemic preconditioning was found to significantly decrease the LDH release after global ischemia, as compared to ischemic preconditioning, thereby further exaggerating the decrease in LDH release caused by ischemic preconditioning. On the other hand, garlic preconditioning was found to significantly increase the LDH release after global ischemia, as compared to the ischemic preconditioned group [Figures 2, 3].

### Effect of preconditioning and garlic extract on ischemic reperfusion induced CK release

**Figure 4:**
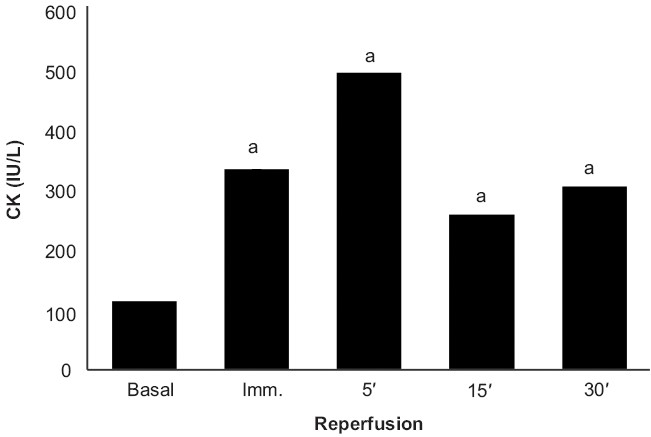
Effect of ischaemia and reperfusion on the release of Creatine Kinase (CK) in isolated rat heart

**Figure 5:**
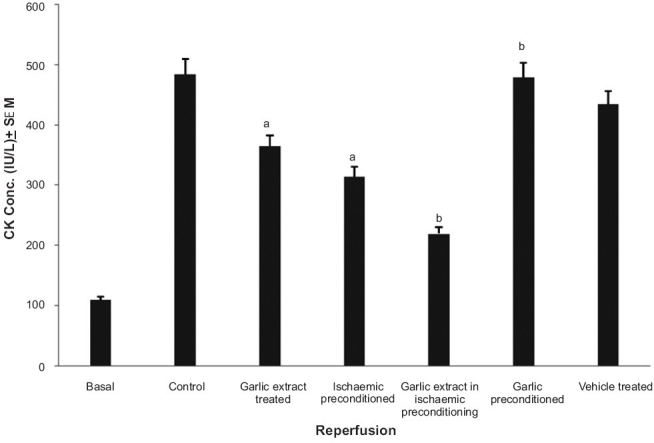
The effect of preconditioning and garlic extract on ischemia reperfusion induced Creatine Kinase (CK) release, after 5 min of reperfusion. a = *P*<0.05 vs basal; b = *P*<0.05 vs control; c = *P*<0.05 vs ischemia preconditioned

The peak release of CK in coronary effluent of isolated rat heart was observed after 5 min of reperfusion [Figure 4]. This is in accordance with previous reports. Ischemic preconditioning and garlic treatment showed significant decrease in CK release after 5 min of global ischemia, as compared to the control group; whereas, garlic preconditioning and vehicle treatment did not significantly alter the profile, as compared to the control. Garlic extract administered during ischemic preconditioning was found to significantly decrease the CK release after global ischemia, as compared to ischemic preconditioning, thereby further exaggerating the decrease in CK release caused by ischemic preconditioning. On the other hand, garlic preconditioning was found to significantly increase the CK release after global ischemia, as compared to the ischemic preconditioned group [Figure 5].

### Effect of preconditioning and garlic extract on myocardial infarct size

**Figure 6:**
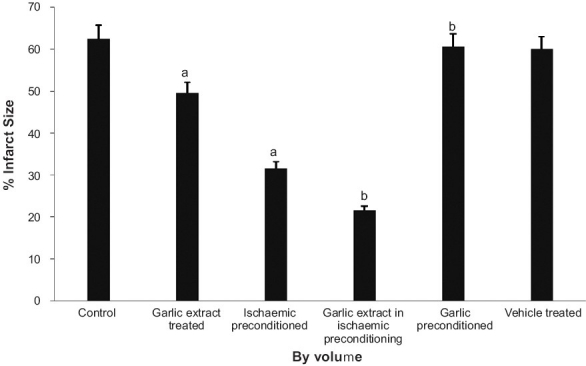
The effect of preconditioning and garlic extract on myocardial infarct size. a = *P*<0.05 control; b = *P*<0.05 vs ischemia preconditioned

The extent of myocardial infarct size in control experiments was recorded to be 70.6 percent th ± 2.5, calculated by volume method [Figure 6]. Ischemic preconditioning and garlic treatment showed significantly reduced myocardial infarct size, as compared to the control group. Whereas, garlic preconditioning and vehicle treatment were not significantly altered. Garlic extract administered during ischemic preconditioning was found to significantly decrease the myocardial infarct size, as compared to ischemic preconditioning, thereby further exaggerating the decrease in infarct size caused by ischemic preconditioning On the other hand, garlic preconditioning was found to significantly increase the myocardial infarct size, as compared to ischemic preconditioned group.

## Discussion

The protocol of four episodes of ischemia interspersed with four episodes of reperfusion, employed in the present study, has been documented to precondition the myocardium.[17] Lactate dehydrogenase (LDH) is a known marker of cardiac injury and the peak release of LDH observed in the present study is immediately and 30 min after reperfusion.[18] It may apparently be suggested that initial release of LDH occurring immediately after reperfusion may be due to ischemic injury and the delayed release of LDH, observed after 30 min of reperfusion, may be due to reperfusion injury. This is also in conformity with the earlier reports.[19–22] Similarly, creatine kinase (CK) is also known to increase in the cardiac injury.[19] Peak release of CK was observed after 5 minutes of reperfusion and is in conformity with the earlier reports.[19,20,22] Ischemia-reperfusion injury has also been quantified by measuring the myocardial infarct size by volume method.[14]

In the present study, an attempt has been made to examine the effect of garlic extract on ischemia reperfusion induced cardiac injury, the effect of garlic on protection offered by ischemic preconditioning and the effect of garlic preconditioning. Garlic extract has been reported to have antiplatelet activity.[23] It inhibits the platelet integrin, group IIb/IIIa, which is the fibrinogen receptor in the platelet surface, and, hence, prevents platelet aggregation and adhesion.[24] It has also been investigated that garlic extract is effective in preventing oxidative stress by reducing the oxidation of lipoproteins tocopherols and ascorbic acid.[25] It also inhibits the enzymes lipooxygenase and cyclooxygenase involved in arachidonic acid metabolism.^[26]^ Garlic preconditioning is not effective in offering cardioprotection, thereby indicating that garlic requires an additional stimulus in the form of ischemia to enhance the cardioprotection offered by preconditioning.

From the above discussion, it may be concluded that administration of garlic extract may prevent ischemia-reperfusion induced myocardial injury, probably by inhibiting platelet aggregation, oxidative stress or by its fibrinolytic properties.
